# Endoscopic-Assisted Curettage of Posterior Proximal Tibia Chondroblastoma: A Case Report and Review of Literature

**DOI:** 10.7759/cureus.52129

**Published:** 2024-01-11

**Authors:** Hawra M Albaqali, Emad Alabsi, Mohammad S Alfehaid, Rasees F Alotaibi, Amani Joudeh

**Affiliations:** 1 Orthopedic Surgery, King Fahad Specialist Hospital, Dammam, SAU; 2 Orthopedics, King Fahd Hospital of the University, Dammam, SAU; 3 Pediatric Neuroradiology, King Fahad Specialist Hospital, Dammam, SAU; 4 Pathology, King Fahad Specialist Hospital, Dammam, SAU

**Keywords:** tumor, pediatric, endoscopic, tibia, chondroblastoma

## Abstract

Chondroblastoma is a benign cartilage-producing bone lesion that characteristically occurs in the epiphyseal region of long bones. The most typical locations are the proximal humerus, proximal femur, distal femur, and proximal tibia. There is no medical treatment for the disease; classically, it must be treated by intralesional curettage and bone grafting.

A 15-year-old female patient presented with chronic knee pain with no antecedent history of trauma. Clinical examination showed deep tenderness on maximum flexion and 15 degrees extension lag with full knee flexion. Plain radiographs and knee MRI showed a lesion in the posterior part of the proximal tibia on the midline, highly suggestive of chondroblastoma. CAT-guided biopsy did not show any evidence of malignancy. Intralesional curettage assisted by endoscopic visualization was done using a small incision, and a bone graft substitute reconstructed the defect.

Endoscopic-assisted curettage of benign bone lesions can be considered in challenging locations with good results.

## Introduction

Chondroblastoma is a rare benign cartilaginous bone tumor; however, it is potentially locally aggressive with a predisposition for the epiphysis of long bones [1]. It represents 1%-2% of all primary bone tumors in young patients [2].

It usually occurs in late childhood or adolescence, and men are more frequently affected, with a ratio of 3-2 [3,4]. The most common locations are the proximal tibia, proximal or distal femur, and proximal humerus [3-5]. As chondroblastoma grows, there will be damage to the epiphyseal plate and the subchondral bone, which will eventually lead to pain, bone deformity, and joint dysfunction [6-8].

Chondroblastoma is characterized by the proliferation of chondroblasts along with areas of mature cartilage, giant cells, and, occasionally, secondary aneurysmal bone cyst formation. While generally regarded as a benign entity, it has an intermediate type of behavior, given its ability to recur locally and, on rare occurrences, metastasize to other sites [2,6,7].

The mainstay of treatment, especially if the growth plate or joint surface is involved, is intralesional curettage with or without the use of adjuvant, such as phenol or hydrogen peroxide, and the bone defects can be filled with bone graft, bone graft substitutes, or bone cement. The treatment has an excellent local control rate with a low recurrence [9,10].

## Case presentation

A 15-year-old female patient, who is otherwise healthy, was referred to the sarcoma unit at the King Fahad Specialist Hospital for further evaluation and management concerning a lesion in the posterior aspect of the left tibia. The patient reported a history of knee pain that is dull, with no antecedent history of trauma. The pain started to affect her walking, and later, she began to limp on the affected extremity. There were no associated constitutional symptoms.

Physical examination showed that the knee was held in 15 degrees of flexion, and there were no localized swelling or skin changes. Palpation showed minimal tenderness at the proximal left tibia. Range of motion showed full flexion but with pain at the extreme range of flexion and an extension lag of 15 degrees both actively and passively. Neurovascular examination is normal, with no local regional lymph node enlargement.

**Figure 1:**
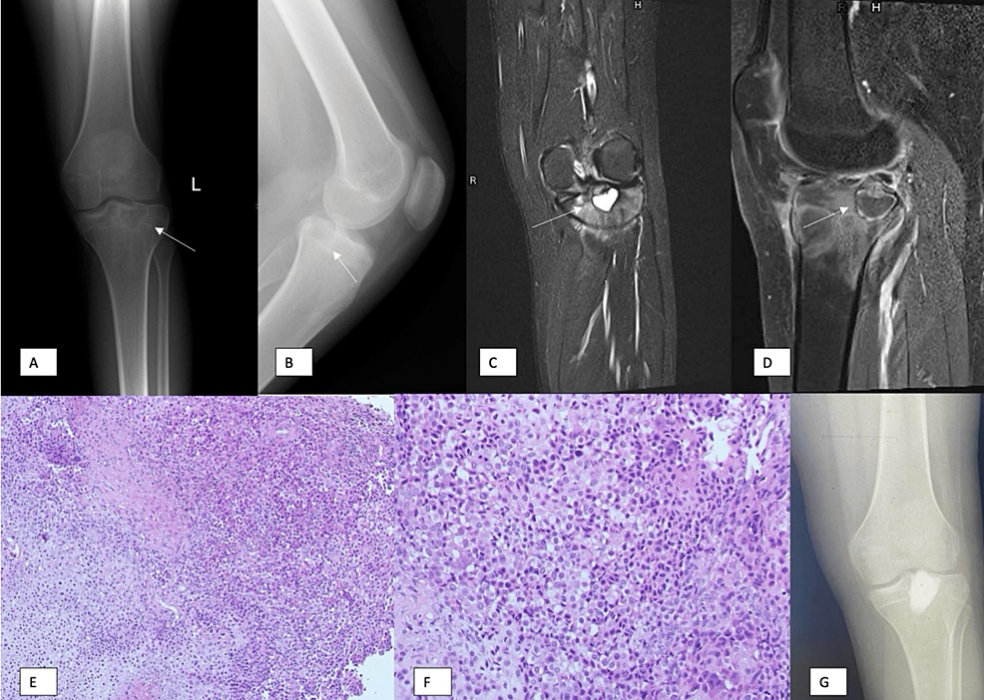
Frontal (A) and lateral (B) left knee radiographs show a well-defined proximal tibial lytic lesion with a sclerotic narrow zone margin centered in the proximal metaphysis. The left knee MRI coronal STIR (C) and sagittal T1 fat-saturated post-contrast image (D) show a well-defined proximal tibial lesion with central T2 hyperintensity and a thin rim of T2 sclerotic hypointensity. There is extensive edema in the surrounding bone marrow and soft tissues. Histopathological assessment (E and F) shows infiltration by round or polyhedral chondroblasts with abundant eosinophilic cytoplasm and well-defined cell borders. The nuclei are oval, hyperlobulated with grooves. Pericellular lace-like or chicken wire-type calcification among degenerative chondroblasts can be seen focally. There is also a chondroid matrix and scattered osteoclast-type giant cells. Postoperative radiographs (G) show the tumor cavity curetted and filled with bone graft substitute. STIR: Short TI inversion recovery.

Plain radiographs of the knee (Figure 1, Panels A and B) showed the lesion, whereas magnetic resonance imaging (MRI) showed the lesion highly suggestive of chondroblastoma (Figure 1, Panels C and D). CT-guided biopsy was done, which showed no evidence of malignancy. So, the constellation of findings is consistent with chondroblastoma.

As the tumor was located in the posterior part of the tibia, the decision was made to proceed with endoscopic curettage and filling with bone graft substitute. It would be challenging to approach open surgery through a posterior approach. The patient and family were consulted, and they agreed with the decision to proceed with this option.

Surgical procedure

**Figure 2:**
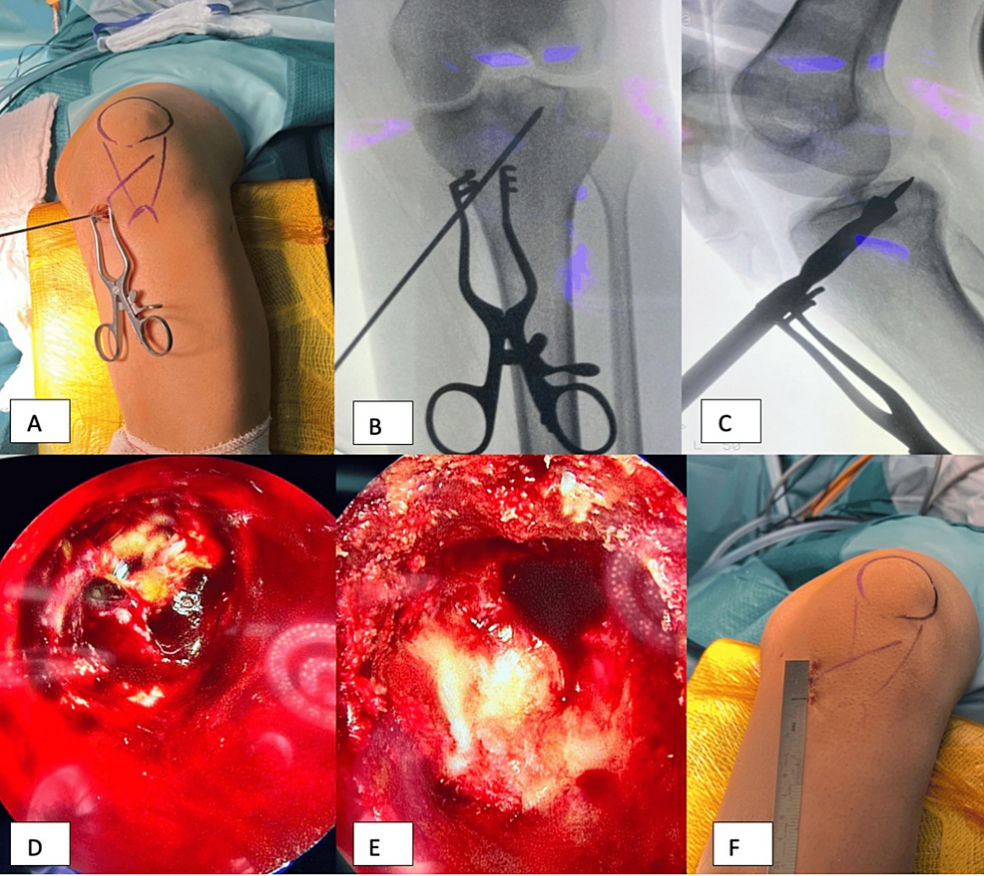
Surgical procedure. (A) Marking of the tibial tuberosity and skin incision. (B) K-wire was inserted and verified on AP and lateral views. (C) K-wire on the lateral radiographs with graduated ACL reamer. (D) Tumor as visualized by the endoscope, with brownish tumor tissue in the cavity. (E) Tumor cavity after curettage using curettes and irrigation; subchondral cartilage is seen on the cavity's roof. (F) Skin incision after closure. AP: Anteroposterior.

The patient was placed in a supine position, under general anesthesia and adductor canal block, for postoperative pain relief. An above-knee tourniquet was applied, and then the lower limb was prepped and draped in the usual surgical manner. Surgery started with lesion localization under fluoroscopic control, and the entry point was determined medially and drawn on the patient skin (Figure 2, Panel A). A 2 mm K-wire was introduced into the lesion starting from the medial proximal tibia and directed to the lesion in the anteroposterior (AP) and lateral positions (Figure 2, Panel B). Once the K-wire reached the center of the tumor, we used the cannulated drill over the K-wire up to the lesion's periphery. We then used the anterior cruciate ligament (ACL) reamers gradually up to size 10 mm to the sclerotic wall of the lesion to gain sufficient access and tunnel size to introduce the endoscope and the curettes (Figure 2, Panel C). The procedure was done under fluoroscopic control to ensure proper tracking and avoid reaming the lesion.

A 30-degree angle endoscope was introduced to visualize the lesion, and variable angle micro-discectomy curettes were used to curette the lesion grossly under adequate visualization by the endoscope (Figure 2, Panel D). With frequent irrigation and curettage, the tumor was cleaned thoroughly and verified by the endoscope. We have also ensured that the overlying cartilage was not penetrated, and the entire tumor was curetted up to bleeding cancellous bone (Figure 2, Panel E). We then utilized argon beam diathermy to cauterize the tumor walls under vision and filled the defect with bone graft substitute, calcium sulfate, and phosphate. C-arm images showed adequate tumor filling, and no extravasation of the bone graft was seen in the joint. The wound was closed in layers, and the skin was closed by subcuticular sutures (Figure 2, Panel F). Tumor samples were sent for the pathology, which showed typical changes of chondroblastoma (Figure 1, Panels E and F). The patient recovered from anesthesia, had a smooth postoperative period, and was discharged the next day in good condition.

## Discussion

Chondroblastoma is a rare benign cartilaginous bone tumor. It is a locally aggressive disease with a predilection for the epiphysis of long bones [1]. It represents 1%-2% of all primary bone tumors, which commonly occur in young patients [2] and males than females [9].

The tumor most commonly occurs in the proximal tibia, followed by the distal femur and the proximal humerus [10,11]. Today, several treatment options are debated for standardized treatment of chondroblastoma; among them, intralesional curettage with or without the use of an adjuvant and filling the cavity with autologous bone graft, allogenic bone graft, bone substitute, or bone cement (PMMA) are the most common surgical techniques reported in the literature [11-13].

The surgical approach depends on the location of the tumor as these tumors can occur in different locations within the bone. In our case, arthroscopic curettage and grafting were used for the treatment of proximal tibial chondroblastoma instead of the conventional open technique as the tumor was located in the most posterior part of the tibia, and access would have been difficult through an open approach. The technique can be done under fluoroscopic control, and curettage with adjuvant can be done under endoscopic visualization.

The advantages of this minimally invasive technique include easy access to difficult locations, less postoperative pain, more cosmetically acceptable wounds, early mobilization, and accelerated rehabilitation. With an adequate tunnel size, the tumor can be curetted using curettes, and high-speed bur and other adjuvant treatments can be used to decrease the local recurrence rate.

## Conclusions

Chondroblastoma is a rare benign tumor that commonly occurs in young people. The mainstay treatment is open intralesional curettage and reconstruction. In our case, we used endoscopic-assisted curettage to gain better visualization of the tumor and better margin resection and prevent risky open procedures, which could lead to many complications. The procedure can be used for treating tumors in difficult locations where open surgery is associated with surgical morbidity.
